# New Appliances and Things Medical

**Published:** 1910-06-11

**Authors:** 


### NEW APPLIANCES AND THINGS MEDICAL.

[We shall be glad to receive at our Office, 28 & 29 Southampton Street, Strand, London, W.C., from the manufacturers, specimens of all new preparations and appliances.]

### RONUK FLOOR POLISH.

The custom which has grown up of recent years of having polished wood floors has drawn attention to the difficulties of their maintenance, and to the disappointment which has very often accompanied their introduction. The great cause of the disappointment has generally been caused by the improper carrying out of the initial preparation. To meet the difficulties of the case the Ronuk Company has invented a sanitary floor polish, the use of which has been adopted by the leading hospitals of the country. The company has also established a Polishing Contract Department, and undertakes the initial preparation of floors and their proper maintenance by a staff of specially trained workmen. Some of the many advantages of the use of Ronuk are that scrubbing and the dampness resulting are dispensed with, the pores of the wood are filled up, and a hard, bright, and durable surface is produced in which germs cannot obtain a harbouring, and the floor can be cleaned with little labour at a small cost. The Ronuk Company has also invented a special staining by which floors of ordinary deal can be converted into polished floors.

The company has just opened a new London depôt at 16 South Molton Street, W., where specimens are on view and where all information with regard to prices and contracts for polishing can be obtained. This new showroom, which was opened recently, is beautifully panelled in oak, and is a fine example of the highest class of English joinery. The floor is laid with oak blocks, and both flooring and panelling have been treated and polished by the company's own staff of workmen, and furnishes a striking testimony to the perfection of finish obtained by the "Ronuk" improved sanitary method of preparing and polishing woodwork. The same applies to the heavy oak beams in the ceiling and to all the wood fittings. The showroom, which is entirely unlike a shop, is furnished with carved oak Jacobean chairs, tables, Persian rugs, with blue and white Delft china on the ledges over the panelling, and the whole effect is extremely artistic and restful. Visitors will be welcomed by the company's representatives, who will be pleased to answer inquiries, and to give every information and advice with regard to the treatment of all kinds of woodwork. The head office and factory of the Ronuk Company is at Portslade, near Brighton, and the company has a depôt at 285 Deansgate, Manchester.

